# Understanding Research Approaches to Assess Sugar-Sweetened Beverage Taxation Policy Implementation and Response in Low- and Middle-Income Countries: Results From a Scoping Review

**DOI:** 10.1093/nutrit/nuaf122

**Published:** 2025-07-21

**Authors:** Payao Phonsuk, Sirinya Phulkerd, Jintana Jankhotkaew, Anne Marie Thow, Penny Farrell

**Affiliations:** Leeder Centre for Health Policy, Economics and Data, Faculty of Medicine and Health, The University of Sydney, Sydney, NSW 2042, Australia; Department of Health Education and Behavioral Sciences, Faculty of Public Health, Mahidol University, Bangkok 10400, Thailand; Institute for Population and Social Research, Mahidol University, Nakhon Pathom 73170, Thailand; International Health Policy Program, Ministry of Public Health, Nonthaburi 11000, Thailand; Leeder Centre for Health Policy, Economics and Data, Faculty of Medicine and Health, The University of Sydney, Sydney, NSW 2042, Australia; School of Public Health, Faculty of Medicine and Health, The University of Sydney, Sydney, NSW 2006, Australia

**Keywords:** SSB taxation, policy implementation, policy response, LMICs

## Abstract

Implementing sugar-sweetened beverage (SSB) taxation policies presents significant challenges in practice, particularly in low- and middle-income countries (LMICs) where resources and capacity are often limited. Expanding the evidence base and knowledge on policy implementation and responses is crucial to identifying key drivers and barriers, as well as developing effective systems for monitoring and evaluation. This study aimed to identify the research approaches used to assess and analyze SSB tax policy implementation and responses at the national level. A scoping review was conducted, drawing on relevant literature from 5 databases—Medline, Web of Science, Scopus, Global Health, and CINAHL—covering publications from 2014 to 2023 inclusive. A total of 24 studies were included in the analysis. Two qualitative studies on policy implementation revealed limitations related to data access, with reliance on publicly available information. The remaining 22 studies, focusing on policy responses, primarily used quantitative methods to evaluate the reactions of industry, retailers, and consumers in terms of price adjustments, purchasing behavior, and consumption patterns. While there is substantial documentation on tax design and structure, enforcement dynamics, stakeholder interactions, and the complexities of policy implementation remain underexplored. This review highlights a critical gap in the research on the multifaceted nature of policy implementation and response. Future studies should prioritize these dynamics and adopt innovative methodologies to enhance the effectiveness of SSB taxation policies and public health interventions in LMICs.

## INTRODUCTION

Noncommunicable diseases (NCDs) are a significant global health concern, particularly in low- and middle-income countries (LMICs), where the burden is rapidly increasing.^1^ One of the major causes of NCDs relates to an unhealthy diet, classified as high in saturated fats, sugar, and sodium.^2^ This dietary pattern is linked to a higher risk of developing obesity, type 2 diabetes, cardiovascular diseases, and other NCDs.^3–5^ In LMICs, specific concerns have been raised regarding the consumption of sugar-sweetened beverages (SSBs), which has been rising steadily due to several factors, including urbanization, increasing incomes, aggressive marketing by the beverage industry, and the availability of cheap, high-calorie drinks.^6^^,^^7^ Sugar-sweetened beverages, which include soft drinks, flavored juices, energy drinks, and sweetened teas, are high in added sugars and calories, contributing to an excessive caloric intake without providing essential nutrients.^8^

One effective NCD preventive policy recommended by the World Health Organization (WHO) is the taxation of SSBs.^9^ This policy approach aims to reduce sugar consumption by increasing the price of these unhealthy products, thereby promoting healthier dietary habits.^10^ Policy implementation in this context refers to the systematic process through which governments translate taxation frameworks into practice via coordinated actions across agencies, enforcement mechanisms, and strategic stakeholder engagement.^11^^,^^12^ However, several challenges impede the effective implementation of SSB taxes in LMICs, limiting their effectiveness. These reflect broader challenges with policy implementation, due to the complexity of the process itself and external factors.^13^ First, many LMICs struggle with limited financial and technical resources necessary for policy enforcement and monitoring.^14^^,^^15^ Second, political will is essential for initiating and maintaining SSB taxes. In many LMICs, policymakers may face opposition or lack the support needed to enact and sustain such policies.^16^^,^^17^ Furthermore, the food and beverage industry wields significant influence and often opposes SSB taxation through lobbying, public relations campaigns, and legal challenges.^17–19^ This resistance can undermine policy efforts and delay implementation.

Another challenge in policy implementation arises from the response of consumers, in which policy can shape their diet behaviors as one of the determinants of health.^20^ Sugar-sweetened beverage taxation policy uses the mechanism of prices to change consumer behaviors. Research indicates that price responsiveness can vary across different consumer groups. For instance, individuals from lower socioeconomic backgrounds tend to reduce their purchases of SSBs more significantly compared with other socioeconomic groups.^21^ Moreover, consumers may respond to prices without awareness of tax.^22^ The important factor in determining the response of price is the pass-through rate, in which the food manufacturers and retailers can pass the burden of tax onto consumers.^23^ These are the mechanisms that influence consumers’ behavior, and which can result in potential success or failure in the SSB tax policy.

To address these challenges and ensure the successful implementation of SSB taxation policies, research plays a crucial role. Empirical studies can provide insights into the best practices, barriers, and facilitators of policy implementation.^24^ This knowledge is vital for designing effective strategies tailored to the unique contexts of LMICs. However, there is a noticeable gap in the literature regarding implementation research approaches specific to SSB taxation in LMICs. Most existing studies focus on the implementation process,^25^ the facilitators and barriers encountered,^14^^,^^25^^,^^26^ price changes,^27^ and the health and economic impacts.^28^^,^^29^ There is a need for comprehensive reviews and updated literature that explore the theoretical frameworks, research tools, and measurement methods used in assessing SSB tax implementation and response.

This scoping review aims to fill this gap by synthesizing current evidence on the research approaches used to assess SSB taxation policy implementation and response in LMICs. Specifically, it seeks to (1) update the literature on SSB taxation policy implementation and response in LMICs; (2) explore the research approaches, including theories, frameworks, tools, and measurement methods used in the policy implementation and response research; (3) identify facilitators and barriers to policy implementation and response; and (4) provide insights into effective strategies for overcoming policy implementation challenges. By addressing these objectives, this review offers valuable guidance for policymakers, researchers, and public health practitioners, ultimately contributing to the enhanced implementation and response of SSB taxation policies and the reduction in NCDs in LMICs.

## METHODS

A scoping review was conducted to review research methods used to assess the implementation and response of SSB taxation policy in LMICs. The search was based on the protocol from the Joanna Briggs Institute.^30^ The following sections outline the steps taken in conducting the scoping review, including the logic model, the search strategy, the study selection process, and data-extraction methods.

### Logic Model to Assess SSB Tax Policy Implementation and Response

**Figure 1.**
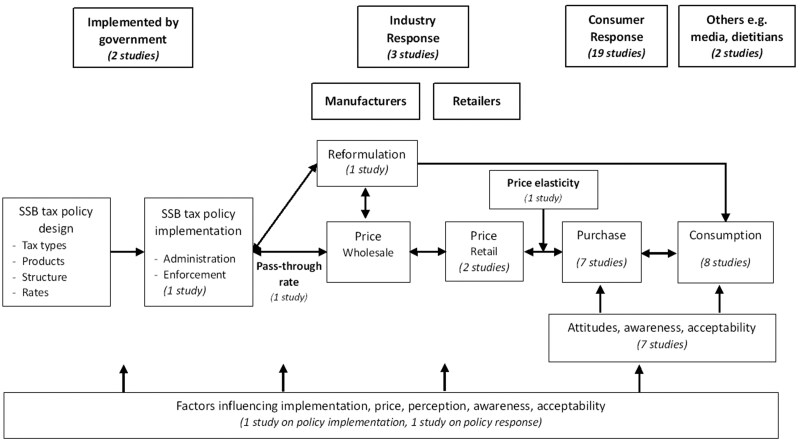
Logic Model to Analyze sugar-sweetened beverage (SSB) Tax Policy Implementation and Response and the Number of Publications on Each Aspect. Three studies assessed more than 1 aspect of policy responses, and no study discussed the change in wholesale price

We used a logic model to establish the key concepts relevant to SSB tax policy implementation and response as the focus for the review search terms and extraction (Figure 1). This model was developed by synthesizing the literature on the pathways of SSB tax policy implementation process and outcomes.^10^^,^^31–34^

The implementation and response of SSB taxation policies involve multiple stakeholders with distinct roles, responsibilities, and interests (Table 1).^31^^,^^35^ Understanding these stakeholder dynamics provides a framework for analyzing how policies are implemented and how different actors respond to them.

**Table 1.** Roles, Responsibilities, and Interests of Key Stakeholders in Sugar-Sweetened Beverage Taxation Policy

Stakeholders	Roles and responsibilities	Interests
Government		
Ministry of Health	Policy development Health-impact monitoring	Public health intervention Cost-effective implementation Health outcomes
Ministry of Finance	Tax administration Revenue collection Monitoring and evaluation	Revenue generation Economic feasibility Cost-effective implementation
Industry		
Manufacturers	Policy compliance Product reformulation	Profit maintenance Market share Brand reputation Consumer loyalty Cost minimization Regulatory compliance
Retailers	Price implementation Sales monitoring	Sales volume Customer retention Profit margins Market competitiveness Consumer satisfaction
Others		
Civil society	Policy advocacy Public awareness Policy monitoring	Public health protection
Research institutions	Evidence generation Policy impact evaluation and assessment Technical support	Evidence-based policy Knowledge translation Research impact
Consumers	Public response Behavior change	Price of the product Availability of choice Test preferences Health awareness

Policy implementation refers to the stage where an established or employed organization translates the policy design into practice.^11^^,^^12^ The implementation of the SSB tax policy begins with the formulation and passage of relevant legislation. The government (eg, Ministry of Health and Ministry of Finance) allocates financial and human resources for policy enforcement and administration. Data and research from previous studies and international examples are utilized to design an effective tax structure, considering tax types, product coverage, structure, and tax rates. Upon enactment, the policy includes clearly defined guidelines for taxable products, tax rates, and compliance requirements.^10^^,^^32^

Policy response refers to the collective actions and reactions of stakeholders, including governments, industries, and the public, in response to the implementation of a policy.^36^ The beverage industry and retailers respond to the tax policy by adjusting their strategies.^37^ Initially, manufacturers may increase wholesale prices of SSBs to pass the cost onto consumers. Some companies may choose to reformulate their products to reduce sugar content, thereby making them exempt from the tax or subject to a lower tax rate.^10^^,^^31^ Retailers must comply with the new pricing structures and ensure that the tax is appropriately applied at the point of sale, potentially increasing retail prices due to higher wholesale costs from manufacturers.^10^^,^^38^

Consumers respond to price increases by altering their purchasing and consumption behaviors.^33^ The degree of this change depends on the price elasticity of demand. Price elasticity is a parameter for estimating the percentage change in SSB demand resulting from a 1% increase in price.^39^ Public and consumer attitudes and awareness also play a critical role in this phase, as informed consumers are more likely to reduce their SSB consumption in favor of healthier alternatives.^26^^,^^32^^,^^40^ The immediate effect is a noticeable decrease in SSB sales and consumption.

The implementation and response to SSB tax policies are influenced by several key factors, including political aspects, stakeholder engagement, and effective policy design.^32^ The tax rate and the extent to which it is passed on to consumers determine its impact on prices and consumption. Consumer attitudes, shaped by awareness of health risks and industry messaging in the media, are crucial for gaining public support. Social acceptability, addressing equity concerns, and the industry's ability to adapt by reformulating products also significantly affect the policy's acceptance and sustainability.^32^

This dynamic interaction between industry and consumers also feeds back into the government's approach to policy implementation. For instance, if the industry significantly reformulates products to reduce sugar content and consumers switch to less-caloric beverages, the government might adjust the tax structure or focus on different products to maintain public health objectives.^41^ However, if consumer awareness and acceptability are low, the government might invest more in public campaigns to improve understanding and support for the tax.^31^ The continuous evaluation of these responses allows the government to adapt and refine the policy, ensuring it effectively promotes healthier consumption patterns and achieves the desired health and economic outcomes. Thus, the logic model captures the ongoing and reciprocal influence among government policies, industry actions, and consumer behaviors in the context of SSB taxation.

### Search Strategy

Peer-reviewed journal articles were selected from the following 5 databases: Medline, Web of Science, Scopus, Global Health, and CINAHL. Key search terms included SSB, taxation, policy, and implementation. Complete details of the search strategy, including specific terms, combinations, and filters for each database, are provided in Table S1. The search strategy was first developed in Medline and then adapted appropriately for other databases while maintaining consistency in concepts and approach. All searches were conducted on November 9, 2023.

### Inclusion and Exclusion Criteria

Peer-reviewed journal articles were included in this review if they (1) were conducted in LMICs, based on the criteria of the World Bank^42^; (2) addressed the SSB taxation policy at the national level; and (3) mentioned the research approach to assess and analyze policy implementation and response. The responses of SSB taxation policy involve any changes in product reformulation or sugar content, prices, pass-through rate, purchases/sales, and consumption. The perception and awareness of the SSB tax policy are also included. We included articles published in English over 10 years, from 2014 to 2023. Publications were excluded if they studied the impact of SSB taxation on health and economic outcomes. Articles focusing on cross-country level analysis were also removed from this study.

### Study Selection, Data Extraction, and Data Analysis

All searched publications from 5 databases were imported to Covidence (Veritas Health Innovation Ltd, Melbourne, VIC, Australia), a systematic review management software, provided by the University of Sydney. The deduplicated documents were removed. Titles and abstracts were screened by 2 researchers (P.P., J.J.) independently following the review protocol. The full texts were selected and assessed based on the inclusion and exclusion criteria. The consensus was reached when there were disagreements during the screening process.

Data were first categorized as the research approach for policy implementation and policy response. Then, we collected data according to the extraction form, created in Microsoft Excel (Microsoft Corporation, Redmond, WA, USA) according to the research objectives. The form followed the logic model (Figure 1), including information about the study (eg, authors, country of study, study objectives), details of SSB taxation policy (eg, type of tax, tax rates, year of implementation), research approach used in the study (theory/framework, study design, tools and measurement, data collection, population), and key findings (including results, limitations of the study, future research). Detailed information on the extraction sheet can be found in Table S2. Each study was assessed using the Preferred Reporting Items for Systematic Reviews and Meta-Analyses extension for Scoping Reviews (PRISMA-ScR) checklist.^43^ As is standard in scoping reviews, a formal quality assessment or critical appraisal of the included studies' methodologies was not conducted, as our primary aim was to map the existing research approaches rather than assess their validity.

## RESULTS

### Study Characteristics

**Figure 2.**
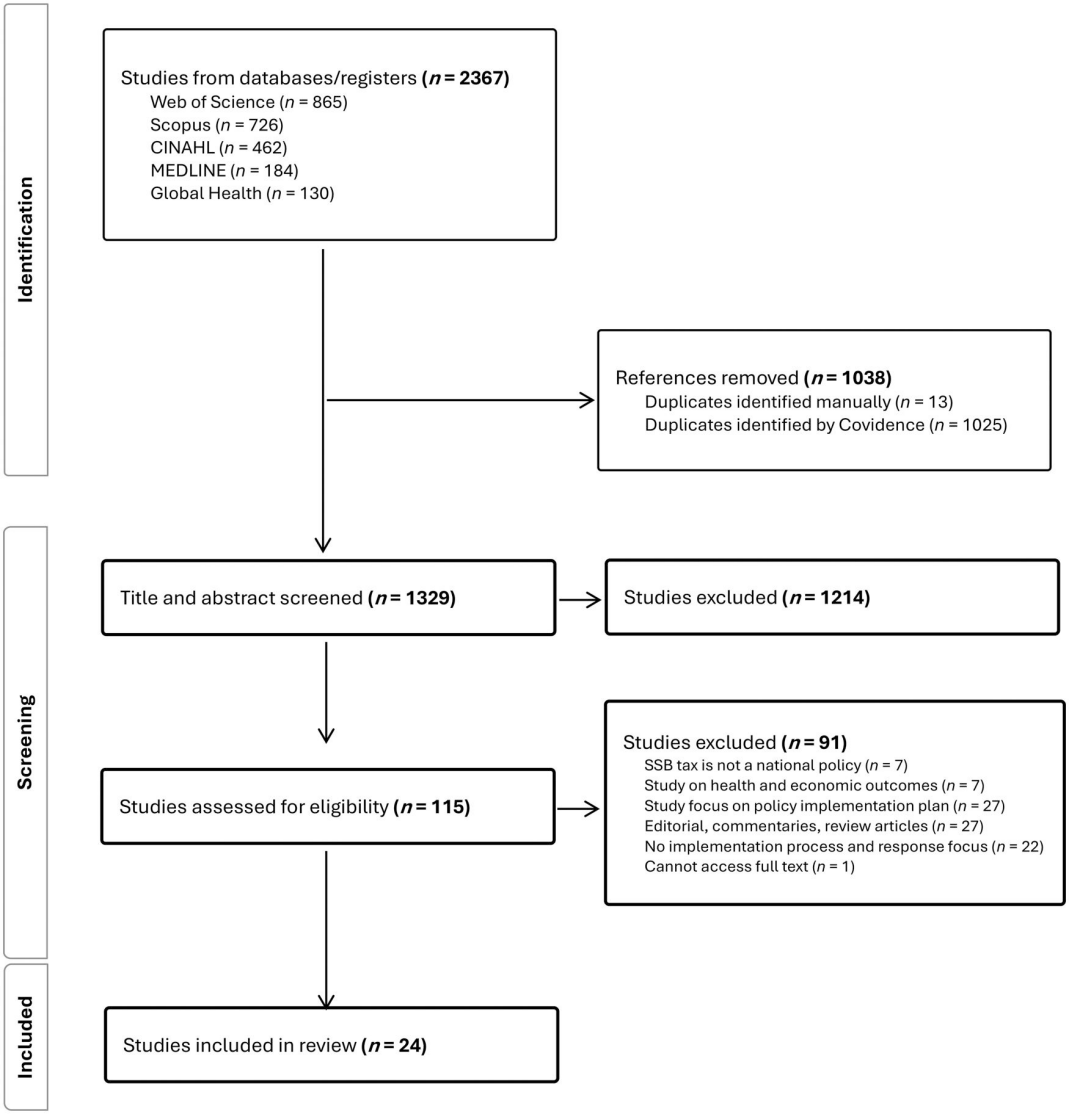
Covidence Flow Diagram of the Article Screening Process. Abbreviation: SSB, sugar-sweetened beverage

A total of 2367 articles from 5 databases were identified. A total of 1038 published studies were removed as duplicates, and the remaining 1329 studies were screened for title and abstract. One hundred fifteen studies were selected for full-text screening. Ninety-one studies were removed according to the criteria, and 24 publications were included for analysis (Figure 2).

Nine studies were from South Africa, 7 were from Mexico, and there were 2 studies each conducted in India and Thailand. Most of the studies (20 publications) applied a quantitative approach to assess policy implementation and response. Two studies focused on the implementation process and factors influencing the implementation. Twenty-two studies analyzed the response towards SSB tax policy implementation. Among these, the focus was on the responsiveness from consumers (19 studies), manufacturers (2 studies), retailers (2 studies), and others such as media (2 studies) (Table 2). Detailed information for all studies is summarized in Table S2.

**Table 2.** Study Characteristics

Country	SSB tax designs					No. of studies in review	Study focus		Expected outcomes	Study design
	Type	Structure	Product coverage	Rate	Year implemented		Implementation	Response		
Ecuador	Excise	Mixed	Nonalcoholic beverages with added sugar	0.18 cents on all sugary beverages with >25 g of sugar per liter	2016	1	—	Consumers^56^	Price elasticity	Quantitative
India	Goods and services tax (GST)	Ad valorem	Nonalcoholic beverages with added sugar and milk with sugar-sweetened or other sweeteners and soya milk drinks	12% on all processed packaged beverages and foods, and 28% GST to be added to aerated beverages and lemonades	2017	2	—	Consumers^61^	Purchasing	Quantitative
								Consumers^52^	Attitude towards policy	Quantitative
Mauritius	Excise	Specific	Nonalcoholic beverages with added sugar	- MUR: 3/100 g of sugar content (2013) - MUR: 6/100 g of sugar content (2016)	2013, revised in 2016	1	—	Consumers^48^	Consumption	Quantitative
Mexico	Excise	Mixed	Nonalcoholic beverages with added sugar	1 peso/L (or 10% tax rate)	2014	7	—	Consumers^62^	Purchasing	Quantitative
								Consumers^57^	Consumption	Quantitative
								Consumers^55^	Purchasing	Quantitative
								Consumers^58^	Consumption Attitude towards policy	Quantitative
								Consumers^46^	Attitude towards policy	Qualitative
								Consumers^59^	Purchasing	Quantitative
								Consumers^63^	Purchasing	Quantitative
Peru	Excise	Ad valorem	Nonalcoholic beverages added sugar, sweeteners and flavorings	25% on drinks with >6 g sugar/100 mL (increase from 17%); 17% on drinks with 0.5–6 g sugar/100 mL (unchanged); 12% on drinks with <0.5 g sugar/100 mL (decrease from 17%)	2018, revised in 2019	1	Process^44^	—	Factors influencing implementation	Qualitative
Philippines	Excise	Specific	Nonalcoholic beverages with added sugar and HFCS, energy drinks	6 pesos/L: drinks using sugar and artificial sweeteners 12 pesos/L: drinks using HFCS	2018	1		Consumers^60^	Consumption	Quantitative
South Africa	Excise	Specific	Nonalcoholic beverages with added sugar, including carbonates (sugar-sweetened and artificially sweetened), concentrates, fruit nectars, sports and energy drinks, and ready-to-drink teas	ZAR 0.021 per gram of sugar over 4 g per 100 mL of nonalcoholic drinks	2018	9	Process^45^	—	Public sentiment	Qualitative
								Dietitians and key industry role players^64^	Attitude toward policy	Quantitative
								Manufacturers Consumers^53^	Reformulation Purchasing	Quantitative
								Consumers^49^	Consumption Attitude towards policy	Quantitative
								Consumers^65^	Attitude toward policy	Quantitative
								Retailers Consumers^50^	Prices Purchasing	Quantitative
								Media^47^	Attitude toward policy	Mixed-methods
								Consumers^51^	Consumption	Quantitative
								Manufacturers Retailers^54^	Prices Pass-through rate Reformulation and price change	Quantitative
Thailand	Excise	Mixed	Nonalcoholic beverages with added sugar	10% on fruit and vegetable juices; 14% on artificial mineral water, soda water, soft drinks with and without sugar or other sweeteners and flavors. Additional specific tax on all drinks with 6–8 g/100 mL – THB 1/L; 8–10 g/100 mL – THB 3/L; >10 g/100 mL – THB 5/L	2017	2	—	Consumers^66^	Consumption	Quantitative
								Consumers^67^	Consumption	Quantitative

Abbreviations: HFCS, high-fructose corn syrup; MUR, Mauritian Rupee; SSB, sugar-sweetened beverage; THB, Thai Baht; ZAR, South African Rand.

### SSB Tax Designs

Among the included studies, Mexico was the first country to implement the SSB tax policy in 2014, followed by Ecuador in 2016. India and Thailand adopted the policy in 2017, while both the Philippines and South Africa implemented it in 2018. Mauritius and Peru were notable for revising their SSB tax policies post-implementation. Mauritius initially implemented the policy in 2013 and revised it in 2016, whereas Peru introduced its tax in 2018 and revised it 1 year later.

Most countries have implemented an excise tax for their SSB taxation policies. South Africa adopted a specific “Health Promotion Levy” approach (9 publications), which considered the sugar content of beverages. India implemented a goods and services tax (GST), while Peru applied a sales tax. Tax rates varied across countries: Ecuador used a fixed tax rate of 0.18 cents, India applied GST rates of 12% and 28%, Mexico imposed 1 peso per liter, and South Africa levied 0.021 rand per gram of sugar exceeding 4 g/mL. Mauritius, Peru, and Thailand had a tiered tax rate, where a higher sugar content had a higher tax rate. The Philippines implemented a higher tax rate for beverages containing high-fructose corn syrup. Most countries applied the tax broadly to all nonalcoholic beverages with added sugars or sweeteners, including carbonated drinks, fruit and vegetable juices, sports and energy drinks, and ready-to-drink tea and coffee. India extended the GST to cover sweetened milk and soya milk as well.

### Research Approaches

#### Policy Implementation

Two studies assessed policy implementation. Both studies applied a qualitative approach, using an in-depth interview and a policy document review. The study in South Africa applied the Multiple Streams Theory as a research framework,^44^ whereas no framework or theory was mentioned in the study from Peru.^45^ Both studies' main target sample group involved stakeholders in the SSB tax policy from government, media, academics, civil society, and industry or industry-related partners. Data were collected at the national level using in-depth interviews and document reviews for analysis.

The research methods used to assess the implementation process had limitations related to data access. Both studies relied solely on publicly available data, which restricted the depth of analysis and may have led to incomplete insights into the implementation dynamics. This lack of access to more comprehensive, internal data sources limited the ability to fully capture stakeholder interactions and enforcement details. Additional data from other relevant institutions, including the industry, were required to complete the data analysis effectively.

#### Policy Response

Twenty-two studies focused on the policy responses. Most studies (20 publications) used a quantitative approach, utilizing both primary and secondary data for analysis. One study adopted a qualitative approach, using in-depth interviews to collect data.^46^ Another study used a mixed-methods approach by applying quantitative content analysis to assess the quantity and quality of content in news media.^47^ None of the studies used a theoretical framework. The main target group of these studies were consumers (19 publications) in different groups, such as teenagers,^46^^,^^48^ people who lived in low-income areas,^49–51^ and patients.^52^ Two studies assessed the industry response^53^^,^^54^ and the other 2 studies evaluated the response from retailers.^50^^,^^54^

Thirteen studies applied secondary data for the analysis. The data were from various sources, including databases published by national statistics institutes such as the National Institute of Statistics and the National Consumer Price Index^54–56^; national health survey repositories^48^^,^^55–60^; commercial market research platforms, such as Nielsen Consumer Panel Service, Europanel, and Kantar-World Panel Division^53^^,^^55^^,^^61–63^; and research databases such as Nexis Uni and ProQuest Central.^47^ Nine studies used data from primary sources, including cross-sectional surveys at both national and city/state levels,^49–52^^,^^64^^,^^65^ longitudinal surveys,^66^^,^^67^ and interviews.^46^

Of the 20 quantitative studies, data were analyzed using various techniques. Statistical descriptive analysis, including mean, percentage, and standard deviation, was used to explain the narrative situation regarding SSB intake, purchasing behavior, and attitudes towards policy implementation.^60^^,^^64^ Mathematical and economic models were used to analyze price elasticity, pass-through rate, and change in sugar concentration in SSB products.^53^^,^^56^ Most studies aimed to explore changes in SSB prices, purchasing, and consumption by comparing the situation before and after tax implementation, using various analysis techniques. Regression analysis, *t* tests, and chi-square tests were commonly used in these studies.^49–52^^,^^54^^,^^55^^,^^57–59^^,^^62^^,^^63^^,^^65–67^ One study applied interrupted time-series analysis to examine the year-on-year growth rate of urban aerated drink purchases. Interrupted time-series analyses typically model the potential impacts of taxes on SSB consumption or purchases as a step change that occurs immediately after the implementation of a tax.^61^ A study in Mauritius utilized a difference-in-differences model to estimate changes in consumption before and after the tax implementation, comparing them with the Maldives as a control country.^48^

Limitations from the studies were found in many aspects regarding their study design and data-collection process. First, several studies reported the absence of substitution effects in their findings.^49^^,^^50^^,^^53^^,^^56^^,^^59^^,^^61^^,^^62^^,^^66^^,^^67^ Substitution effects refer to the change in demand for a product when there is a change in its price.^68^ In the SSB tax policy case, substitution products can be alternative beverages such as water or locally made products. Second, the samples included in several studies were not generalizable to or representative of the whole population.^46^^,^^51^^,^^52^^,^^62^^,^^64^^,^^65^ Third, the study design had several limitations, including the use of a cross-sectional approach, which did not allow for the exploration of changes in purchasing or consumption over time, and the absence of a pre-post implementation effect design.^48^^,^^50^^,^^54^^,^^58^ The use of secondary data also posed challenges in terms of data coverage, particularly regarding the geographic areas, products, and types of stores included in the study.^55^^,^^59^^,^^61^^,^^63^ Due to limited data, some studies reported that they could not identify a causal relationship between the SSB tax and changes in behavior.^47^^,^^49^^,^^53^ Finally, when data were collected at the individual level, challenges such as recall bias,^60^^,^^66^^,^^67^ underreporting of consumption,^57^^,^^58^ self-selection bias,^64^ and fatigue^65^ may have resulted in inaccurate data.

### Key Findings From SSB Tax Policy Implementation Analysis

#### Implementation by Government

All studies discussed the design and structure of the SSB tax enacted at the national level. However, none of the studies addressed the administrative processes involved. One study from South Africa implicitly addressed policy enforcement by examining public and stakeholder sentiments toward the SSB tax. News articles following the implementation of tax, primarily originating from the sugar industry, highlighted concerns about imported cheap sugar, duty-free exemptions, and declining sugar prices. These economic concerns echoed industry arguments in South Africa, where the industry successfully lobbied against certain aspects of the tax by raising fears of unemployment and economic harm. Such opposition may have contributed to the failure to revise the tax during the 2020–2021 fiscal year, where industry pressure led to modifications favoring stakeholders and stalled regular tax adjustments.^45^

#### Factors Influencing Policy Implementation

The studies from Peru and South Africa illustrated that the implementation of SSB taxation policy involves a complex interplay of facilitators and barriers. In Peru, strong political leadership and commitment significantly facilitated the adoption of health taxes. Leaders who prioritized public health and engaged with stakeholders, including civil society organizations and public health advocates, were pivotal in advancing the tax policies. Economic arguments emphasizing the burden of NCDs and the potential revenue for health initiatives also played a critical role in garnering support. However, the process was hindered by political opposition and resistance from industry groups, coupled with concerns about potential economic impacts on businesses and consumers. Negotiation challenges further complicated the implementation process due to the need to reconcile diverse interests among stakeholders.^44^

Similarly, in South Africa, the adoption of the SSB tax was facilitated by the recognition of the obesity epidemic and the strong evidence supporting the effectiveness of such taxes in other contexts. Political support, particularly from key figures aligning the tax with broader public health goals, was crucial in overcoming resistance. Nonetheless, industry opposition, which included significant lobbying efforts and public campaigns, posed substantial barriers. Additionally, logistical challenges related to tax collection, enforcement, and public awareness required considerable effort and resources to address effectively.^45^

### Key Findings From SSB Tax Policy Response Analysis

#### Industry’s, Consumers’, and Others’ Responses

According to the logic model (Figure 1), data were categorized based on actors’ responses to SSB tax policy. Manufacturers and industry typically responded with reformulation^53^ and pass-through rate adjustment,^54^ while retailers focused on price changes.^50^^,^^54^ Consumers’ responses included price elasticity,^56^ changes in purchasing,^50^^,^^53^^,^^55^^,^^59^^,^^61–63^ consumption,^48^^,^^49^^,^^51^^,^^57^^,^^58^^,^^60^^,^^66^^,^^67^ and attitude towards SSB tax policy.^46^^,^^49^^,^^52^^,^^58^^,^^65^ Media^47^ and health professionals, such as dietitians,^64^ also expressed attitudes on policy implementation.

A notable study from South Africa reported an increase in lower-sugar reformulated beverages from 5.2% to 17.1% post–tax implementation reducing sugar intake by 4.9 g per capita per day.^53^ Additionally, the pass-through rate was estimated at 68%, with increased SSB prices reported.^54^ A study in Ecuador found that a 10% tax-induced price increase led to a 13.5% decrease in soft drink consumption.^56^

Six studies reported reductions in SSB purchases, ranging from 6.3% to 40%, with the greatest decreases among low-income households and small retail outlets.^55^^,^^59^^,^^61–63^ A study in Mexico focused on income levels and found a significant decrease in SSB purchases among lower-income groups. In South Africa, a study indicated that the reformulation of sugar content led to a reduction in purchasing.^53^ Additionally, the greatest decreases in SSB purchases were observed in stores such as supermarkets and small, locally owned shops.^50^

Most studies on consumption found declines following tax implementation,^49^^,^^51^^,^^57^^,^^58^^,^^60^^,^^67^ although a study in Mauritius showed an increase in consumption in teenagers.^48^ Attitudes toward the tax varied; some studies from India and Mexico suggested higher taxes would lead to reduced consumption and preference for homemade products.^46^^,^^52^ Awareness of the tax played a key role in changing purchasing behavior,^65^ but awareness among health professionals remained low.^64^ Two studies from Mexico and South Africa identified a link between SSB tax awareness and a reduction in consumption.^49^^,^^58^ Media coverage was mixed, with support driven by health concerns and opposition focusing on economic impacts.^47^

#### Factors Influencing Policy Response

Our review identified several facilitators and barriers influencing the success of SSB tax policies. Key facilitators included increased public awareness and understanding, which positively impacted compliance and reduced SSB consumption.^58^^,^^65^ Psychological factors, such as heightened awareness and intentions to reduce intake, further enhanced policy effectiveness in terms of health outcomes, particularly reductions in sugar consumption and related metabolic disease risk.^49^^,^^52^ Economic mechanisms, such as changes in SSB prices, also played a critical role, prompting consumers to reduce consumption or switch to healthier alternatives such as bottled water.^46^^,^^50^^,^^56^^,^^59^^,^^62^^,^^63^

Barriers to policy response included limited knowledge about the tax and the risks of high sugar consumption, which negatively affected acceptance in countries such as South Africa and Thailand.^65^^,^^66^ Sociodemographic factors, such as age, gender, education, and socioeconomic status, also shaped responses, with younger females and less-educated individuals showing less support for the tax.^52^^,^^65^ Personal preferences for taste and habitual consumption were found to limit the tax's impact.^46^ Furthermore, industry opposition, amplified by media campaigns highlighting economic concerns such as job losses, also posed a significant barrier to policy implementation, complicating public acceptance and legitimacy.^47^

## DISCUSSION

This scoping review provides a comprehensive assessment of research approaches used to assess SSB taxation policy implementation and response in LMICs. Our review identified a clear distinction between the focus areas of existing research on SSB taxation policy. Only 2 studies on policy implementation processes were included in this review. The findings highlighted the importance of stakeholder engagement, emphasizing the key governmental stakeholders such as Peru’s Ministry of Finance, which has primary authority in taxation matters.^44^ The review also revealed detailed enforcement strategies and sentiments towards policy using media, which are crucial to reflect public opinion on policy implementation.^45^ Research on SSB tax policy response primarily examines changes in consumer behavior and industry adaptations to the tax policy. The studies frequently utilized secondary data from national, company, and research databases to estimate the effects of SSB taxes on consumption patterns and public health outcomes.

The findings reflect a disparity in the volume of research on policy implementation compared with policy response. Overall, the literature on policy implementation when compared with the literature on policy response presents significant methodological challenges. These were related to challenges with systematically studying the factors related to policy implementation due to the complexity of the implementation process. Implementation is multilayered, involving the coordination of government agencies, local authorities, industry stakeholders, and public communication efforts.^12^ This complexity makes it more difficult to study than the relatively straightforward measurement of policy outcomes, such as changes in consumption or health outcomes.^69^ Moreover, the implementation process of SSB taxation policy varies widely based on local context, political environment, administrative capacity, and stakeholder involvement.^70^ These factors introduce variability that complicates the generalizability of findings across different settings and increases the complexity of policy implementation studies in this area.

Another challenge highlighted by this review was data accessibility in studying SSB tax policy implementation. Unlike conventional population-based public health data, which are often more accessible, detailed records of policy implementation processes—such as internal government communications, negotiations with stakeholders, and enforcement actions—are typically not publicly available or systematically documented. Furthermore, certain aspects of the implementation process may be confidential, making it difficult for researchers to obtain comprehensive data needed to fully understand implementation dynamics.

There is a notable gap in research concerning the dynamics of policy implementation, particularly how government and industry interact with each other and respond to consumer behaviors. Industry is a crucial stakeholder in SSB taxation policy, significantly influencing both its design and implementation. Beverage manufacturers and distributors can shape the policy through lobbying, public relations efforts, and pricing strategies.^71^ Their responses, such as price adjustments or legal challenges, can directly impact the policy's effectiveness, making their role vital to the success of SSB taxes.^70^^,^^72^ The process of implementing policies, such as SSB taxes, is not merely a top-down exercise; it involves a complex interplay between various stakeholders.^12^ Companies may form public–private partnerships, present themselves as key contributors to national policy, seek out loopholes, or engage in public campaigns to sway consumer opinion,^31^^,^^73^ while governments may need to negotiate with companies to ensure compliance and encourage product reformulation.^74^ Consumer behaviors and attitudes are further shaped by both government communication strategies and industry actions, such as price adjustments or marketing tactics.^31^ The processes governments and industry follow to roll out policy—through public messaging, enforcement measures, and stakeholder engagement—can significantly shape consumer impacts.^10^ Despite the importance of these dynamics, there is limited research exploring how these interactions unfold in practice, leaving a critical gap in our understanding of the full implementation process.

The findings of this scoping review highlight several key facilitators and barriers to the implementation and response of SSB taxation policy. Strong political commitment, particularly from high-ranking government officials and key institutions, is a crucial driver for the adoption and sustained implementation of SSB taxation policies. In countries such as Mexico and South Africa, the Ministry of Finance and Ministry of Health, alongside public health advocates and consumer support groups, played a pivotal role in championing the SSB tax by positioning it as a public health priority.^75–77^ Evidence-based policymaking also serves as a vital facilitator, with successful implementation often relying on robust scientific evidence that highlights the link between sugary drink consumption and NCDs, such as obesity and diabetes. This evidence strengthens the case for SSB taxation by framing it as a necessary public health intervention.^78^^,^^79^ Public awareness and acceptance further enhance policy implementation. Targeted public health campaigns, such as those seen in Mexico, have raised awareness about the risks of SSB consumption and fostered public support for the tax, demonstrating how effective communication can shift public perception.^75^ Additionally, the economic mechanism of SSB taxation, which increases the price of SSB, has been shown to reduce both purchases and consumption.^31^^,^^32^ When these facilitators are aligned, they create a supportive environment that fosters the successful implementation and public acceptance of SSB taxation policies, ultimately contributing to the achievement of the policy’s objectives.

Multiple important barriers exist to the effective implementation and response to SSB tax policies described by the studies included in our review. Industry influence poses a significant barrier, as beverage manufacturers and distributors often engage in lobbying and media campaigns to oppose tax measures or cast doubt on their health benefits.^80^^,^^81^ These efforts can undermine the effectiveness of SSB taxation policies by shaping public perception and influencing political decision-making, as seen in Mexico and Brazil.^82^^,^^83^ These industry efforts can include legal challenges, aggressive marketing strategies, and price adjustments designed to mitigate the tax’s impact on consumption.^80^ Additionally, a lack of knowledge and awareness among both policymakers and the public about the health risks of sugary beverages and the potential efficacy of taxation further impedes policy adoption and compliance. Without a clear understanding of how SSB taxes can positively impact public health, resistance to such measures may persist.^78^

This study reviews the aspects relating to the assessment of SSB tax policy implementation and response. However, several novel research questions and approaches that could enhance the assessment of SSB tax policies can be identified. A systems thinking approach is recommended to understand the broader implications of SSB taxation, including shifts in consumption patterns, market adaptations, and changes in social norms around sweetened beverages.^72^ Additionally, future research should explore the long-term effects of SSB taxation on public health and economic outcomes, considering variables such as substitution effects and market adaptations.^84^

The critical importance of rigorous research in informing SSB tax policy implementation and response has been highlighted in this review. Research on SSB tax policy implementation and response has the potential to help establish standards for methods and tools used in policy analysis, ensuring consistency and reliability in findings.^70^ Understanding the implementation process can inform the best practices and identify potential challenges, leading to more effective policy design and enforcement.^15^ Additionally, assessing the policy response provides evidence of the real-world impacts of SSB taxes, such as behavioral change and health outcomes, guiding future public health interventions.^85^ Therefore, establishing standardized methods and tools for assessing these policies is crucial for generating reliable and comparable data across different contexts. This standardization will facilitate the development of evidence-based policies that effectively reduce SSB consumption and improve public health outcomes in LMICs.

The implementation of SSB taxation policies is particularly crucial in LMICs, where SSB consumption, especially soft drinks, has reached concerning levels.^4^ These countries are experiencing a rapid nutrition transition characterized by increasing consumption of ultra-processed foods and beverages, with SSBs being one of the primary contributors to excess sugar intake.^86^^,^^87^ The increasing burden of NCDs, limited healthcare resources, and aggressive marketing practices by beverage companies in these emerging markets underscore the urgency of implementing effective SSB taxation in LMICs. For instance, studies from Mexico, South Africa, and Thailand, where SSB consumption was notably high, demonstrated significant reductions in purchases and consumption following tax implementation, particularly among lower-income populations.^21^^,^^50^^,^^67^^,^^88^ This suggests that SSB taxes can be an especially powerful tool for public health intervention in LMICs, where the economic burden of NCDs can have devastating effects on household finances and national healthcare systems.^89^ Therefore, understanding the implementation dynamics and response patterns to SSB taxation in these contexts is crucial for maximizing policy effectiveness and protecting vulnerable populations from the health and economic consequences of excessive SSB consumption.

This review has several limitations. First, the literature search was restricted to English-language publications, which may have excluded relevant studies published in other languages. Second, by following current World Bank country classifications for LMIC status,^42^ our review may have excluded valuable implementation experiences from countries that were LMICs at the time of their SSB tax implementation but have since transitioned to high-income status. Additionally, the review focuses exclusively on single-country case studies, limiting its scope and excluding research that explores cross-country comparisons or broader insights from other LMICs. These constraints may affect the generalizability of the findings across different contexts.

A notable limitation in evaluating SSB taxation effectiveness is potential publication bias. Studies demonstrating significant reductions in SSB consumption or successful implementation may be more likely to be published than those showing null or negative results, potentially overestimating policy effectiveness. While our review identified successful policies in countries such as Mexico or South Africa,^51^^,^^63^ unpublished evaluations from other LMICs where implementation faced significant challenges or failed to achieve intended outcomes may exist. This bias could particularly affect the understanding of implementation barriers in resource-limited settings where evaluation capacity may be constrained.^14^ To address this, future research should prioritize documenting both successful and unsuccessful policy implementations through mandatory evaluation protocols, providing policymakers with a more comprehensive evidence base for decision-making in LMIC contexts.^70^ Finally, our analysis did not systematically assess potential industry funding or conflicts of interest in the included studies. This could provide important context for interpreting findings, as funding sources may influence study design, analytical approaches, or reporting of policy impacts. Future research should incorporate analysis of funding patterns and potential conflicts of interest to strengthen the evaluation of evidence in this field.

## CONCLUSION

This review examined the complexities and challenges of implementing SSB tax policies, emphasizing the multifaceted nature of policy implementation and response through various research approaches. While the effectiveness of SSB taxes in changing prices and consumer behavior is well documented, the implementation process is less understood and poses significant difficulties for study. The crucial role of industry in shaping these policies is acknowledged, yet the interactions between government, industry, and consumers during implementation are not thoroughly explored. This gap highlights the need for more research on these dynamics to better understand their impact on policy effectiveness. The literature also indicates a research preference for measurable outcomes, such as changes in SSB prices and consumption, due to the ease of quantification and the immediate availability of data. To advance the field, future research should focus on a more in-depth examination of the implementation process, drawing on innovative research approaches. This will provide policymakers with a comprehensive understanding of the factors that determine the success or failure of SSB tax policies, supporting the development of more effective and context-sensitive interventions that could be applied to other food and nutrition policies.

## Supplementary Material

nuaf122_Supplementary_Data

## Data Availability

This article contains all the original findings from the research. Inquiries can be directed to the corresponding author.
