# Supplementary material for: Understanding Research Approaches to Assess Sugar-Sweetened Beverage Taxation Policy Implementation and Response in Low- and Middle-Income Countries: Results From a Scoping Review
Source: Nutr Rev. 2025 Jul 21;84(6):1290–304. doi: 10.1093/nutrit/nuaf122 (PMC13161759; doi:10.1093/nutrit/nuaf122)
Supplement: nuaf122_Supplementary_Data [file nuaf122_supplementary_data.docx]

**Supporting Information**

**Table S1: Search strategy and search terms**

| **Databases** | **Search date** | **Specific search terms** | **Filters** | **Number of papers** |
| --- | --- | --- | --- | --- |
| **Medline** | 9/11/2023 | (policy/ or health policy/ or nutrition policy/ or regulat*.mp. or legislat*.mp. or law*.mp. or guideline.mp. or action plan*.mp.) AND (implement* or enforc* or adapt* or adopt* or appl*) AND (beverages/ or carbonated beverages/ or coffee/ or energy drinks/ or sugar-sweetened beverages/ or tea/ or (drink* or beverage* or fizzy or sugar* or sweetened or soda or cola or coke or carbonated).mp.) AND (Taxes/ or (tariff* or tax or taxes or taxation or excis* or duty or duties or levy or levies).ti,kw.) | - Years: 2014-2023 - Language: English - Publication type: Article | 184 |
| **Web of Science** | 9/11/2023 | (Health Policy or Policy or Nutrition Policy or Food policy or regulat* or legislat* or law) AND (implement* or enforc* or adapt* or adopt* or appl*) AND (beverage* or drink* or carbonat* or sugar* or sugar sweetened or fizzy or soda) AND (tax* or excis* or fiscal) | - Years: 2014-2023 - Language: English - Document type: Article | 865 |
| **Scopus** | 9/11/2023 | ((health OR nutrition OR food) AND policy) OR policy OR legislat* OR regulat* OR law) AND (implement* OR enforc* OR adapt* OR appl*) AND (beverage* OR carbonated OR drink* OR sugar-sweetened OR fizzy OR sugar* OR soda ) AND (tariff* OR tax* OR excis* OR dut* OR lev* OR fiscal) | - Years: 2014-2023 - Language: English - Document type: Article | 726 |
| **Global Health** | 9/11/2023 | (policy/ or food policy/ or health policy/ or nutrition policy/ or regulat*.mp. or legislat*.mp. or law/) AND ( implement*.mp or law enforcement/ or enforc*.mp. or adapt*.mp. or adopt*.mp. or appl*.mp.) AND (soft drinks/ or beverages/ or cola beverages/ or sugar sweetened beverages/ AND taxes/ or fiscal policy) | - Years: 2014-2023 - Language: English - Publication type: Article | 130 |
| **CINAHL** | 9/11/2023 | TX (Health Policy or Policy or Nutrition Policy or Food policy or regulat* or legislat* or law ) AND TX ( implement* or enforc* or adapt* or adopt* or appl* ) AND TX ( beverage* OR carbonated OR sugar-sweetened OR fizzy OR soda ) AND ( tax* OR excis* OR fiscal ) | - Years: 2014-2023 - Language: English - Peer-reviewed | 462 |
| **All papers** | | |  | **2,367** |

**Table S2: Detailed information on studies**

| **Author** | **Country** | **Focus of study** | | **Study design** | **Theory/ Framework** | **Target sample** | **Data** | | | | **Data collection/ analysis** | **Key findings** | **Study limitation** | **Suggested future study** |
| --- | --- | --- | --- | --- | --- | --- | --- | --- | --- | --- | --- | --- | --- | --- |
|  |  | **Imple mentation** | **Response** |  |  |  | **Input data** | **Source** | **Level of data** | **Primary (P)/ Secondary (S)** |  |  |  |  |
| Segovia et al., 2020 | Ecuador |  | **/** | Quantitative | N/A | Household (purchasing data) | Household income and expenditure, SSB purchases | National Survey of Income and Expenditure for Urban and Rural Households and National Institute of Statistics and Census | National | S | 5 non-alcoholic beverages were analyzed (3 for sugary, 2 for non-sugary). Price elasticity analysis was performed using a demand system extension, called QUAIDS. | A 20% increase in the price of SSBs will decrease the consumption of soft drinks and other sugary drinks by 27% and 22%, respectively. Soft drinks have own price elasticity of -1.35. | Only at-home consump-tion was estimated - Did not include other substitution or complementarity patterns beyond beverages | - |
| Mandal et al., 2020 | India |  | **/** | Quantitative | N/A | Patients and accompanying persons visiting public hospital | Awareness and perceptions regarding taxes on SSB | Cross-sectional survey (Interviewer-administered questionnaires) | City | P | The questions were about sociodemographic details, awareness and perceptions regarding taxes on SSBs. Data were analyzed using a descriptive approach. Univariable logistic regression analysis was conducted to find out the factors predicting participants' favorable response to increase of taxes present on SSBs | 75.1% of the sample were not aware of tax. 66.2% agreed that they would decrease consumption if taxes were increased, and 81.9% agreed that they would prefer homemade beverages after a tax increment. | Majority of sample were males, with missing female participants | Study on a qualitative approach to understand tax preference among various groups of population |
| Law et al., 2021 | India |  | **/** | Quantitative | N/A | Household (purchasing data) | Monthly total volume of aerated drink purchases at household level in 15 states | Kantar-World panel Division, India | State level | S | The study applied interrupted time series (ITS) analysis of year-on-year growth rate of urban aerated drink purchases. | The percentage changes in average monthly purchases ranged from 24%-40% overall. ITS analysis also found a negative change in the year-on-year growth rate of state-level monthly aerated drink purchases. | - Did not find sufficiently detailed state-level information on the pre-GST tax levels of aerated drinks for the study period - The dataset does not cover purchases in rural areas - The dataset cover only take-home purchases - Did not have detailed data on purchases of caffeinated beverages, water or natural juices (substitutional effects) | Study that identify state-specific effects of the GST on changes in the tax system and prices |
| Cawley et al., 2022 | Mauritius |  | **/** | Quantitative | N/A | Youths aged 12-17 years | SSB consumption | The Global School-Based Student Health Survey | National | S | Data were analyzed using the difference-in-difference model to estimate consumption before and after tax, and between the country (taking Maldives as a comparison country). | The tax was associated with an increase in SSB consumption of 0.109 drinks per day. However, models estimated separately by sex indicate that the probability that boys consumed SSB fell by 9.4 percentage points (11%). | Data from cross-sectional study, cannot observe change over time | Study on the prices of SSB and where youth get SSB from |
| Salgado Hernández et al., 2023 | Mexico |  | **/** | Quantitative | N/A | Household (purchasing data) | SSB prices and purchasing | Nielsen Consumer Panel Services | City (urban setting) | S | Data on SSB household purchases and SSB prices in 2012–2015 were retrieved from the Nielsen Mexico Consumer Panel Service (Nielsen CPS). SSB purchases before and after tax implementation were assessed through a price-tertile stratified linear regression model. | The study found a statistically significant purchase reduction ranging between 10.80 and 13.79 ml/capita/day across taxed beverages from the middle-price SSB after the tax implementation. There were no significant changes in the low- and high-price tertiles. | - Data were not generalised  - Did not analyze purchase changes in specific combinations of producers, brands, and package sizes - Did not assess the substitution of SSB for untaxed beverages | Study to test the double tax amount and its effect on purchasing |
| Sánchez-Romero et al., 2020 | Mexico |  | **/** | Quantitative | N/A | People aged more than 19, and complete beverage consumption information | Soft drink consumption before- and after-tax implementation in 3 waves of the cohort study (2004-06, 2010-13, and 2017-18) | Health Workers Cohort Study | City | S | Data were analyzed using descriptive statistics (mean and standard deviation) and a logistic regression model (OLCRE) to evaluate the association of tax and soft drink consumption. | Tax was associated with a 6.8 and 6.1 percentage point decrease in a group with medium and high soft drink consumption, respectively, after the tax implementation | - No comparison group - Sample included health workers who may be aware of the effect of SSB and underreport SSB consumption | Study identifies the long-term implications of changes in body weight and related diseases |
| Pedraza et al., 2019 | Mexico |  | **/** | Quantitative | N/A | Household (purchasing data) | 1. Household-level purchasing  2. Nutrition information data | 1. Nielsen database  2. UNC Mexican Nutrition Fact Panel (MxNFP) dataset | National | S | Weighted and adjusted means for volume, calories, and sugar at the household-monthly level, in overall and by store type, were analyzed using logistic regression. | Volume of taxed purchases decreased by 30 ml in year 1 and 2 of tax implementation, calories and sugar from taxed beverages were also decreased (11% reduction from year 1 to year 2) | - Using the same nutritional profile for both pre-tax and post-tax periods may not reflect any reformulation that may occur - Limited by Nielsen data on products in store, could not capture other sources of beverage purchasing within the household - Purchasing data could not verify real consumption | - Study in different SES to understand the socioeconomic determinants of the tax effect and see the differences across store types. - Study on annual changes in the taxation status of beverages and their nutritional content to better understand the tax's effects on manufacturing, purchasing, and consumption each year after implementa-tion. |
| Alvarez-Sanchez et al., 2018 | Mexico |  | **/** | Quantitative | N/A | Adults aged 20-59 | SSB consumption, tax awareness, psychosocial and environmental determinants of SSB consumption | National Health and Nutrition Survey (ENSANUT) | National | S | Data were collected from the 2016 Mexican National Health and Nutrition Survey (ENSANUT). The questionnaire was from the Perception of Obesity, the Physical Activity and Diet Questionnaire. FFQ was conducted to collect intake data. Chi-square and logistic regression were performed for data analysis. | 65.2% of sample reported being aware of the SSB tax. Those who were aware of the SSB tax were more likely (OR = 1.30) to report a decrease in SSB consumption. Self-efficacy and liking of SSBs were significantly associated with a reported decrease in consumption and with current consumption. | - Data were self-reported - Did not apply pre-post design, unable to assess change in measures before and after the SSB tax.  - Effect of other public health intervention on decreasing SSB consumption | Study to understand the signalling effect of taxes and the publicity of taxes on SSB consumption |
| Ortega-Avila et al., 2018 | Mexico |  | **/** | Qualitative | N/A | Adolescents | Semi-structured interviews | Interviews | City | P | Participants were recruited through an online survey conducted in the previous study. Samples were purposively selected as they consumed at least one portion of SSB daily. The researcher developed the interview questions with the objective to explore factors associated with SSB intake and perception of SSB taxation. Data were analyzed using a thematic approach. | The sample was unaware of SSB taxation. Participants mostly perceived that the SSB tax would not affect their SSB consumption patterns and their decision to buy SSB. They thought that a 10% increase in price was too small and did not affect their intake. Taste preferences and addition were important drivers of participants’ SSB purchases. If SSB prices were to increase further via a higher tax, participants would consider substituting SSB with other beverages, such as home-made drinks (e.g. 100 % fruit juices and aguas frescas), non-caloric instant-flavoured drinks and water. | - Participants came from high income background, preventing generalization of findings to low-income group | - |
| Colchero et al., 2017 | Mexico |  | **/** | Quantitative | N/A | Household (purchasing data) | Beverages purchasing, expenditure on food and beverages | National Income and Expenditure Survey | National | S | Changes in purchases in per capita liters per week were estimated with the use of 2-part models (regression analysis) to adjust for nonpurchases. The dependent variable was weekly purchases of beverages (SSBs or water) in per capita liters per week, which was estimated by dividing for each beverage the total liters purchased by the household by the number of members of the household. | The study found a 6.3% reduction in the observed purchases of SSBs in 2014 compared with the expected purchases in that same year based on trends from 2008 to 2012. The reduction was high in lower income group, residents in urban areas and households with children. The study also found a 16.2% increase in bottled water purchases. | - Taxed and untaxed beverages could not be differentiated due to limitation of survey data - Consumption of potable water or any homemade beverage was not reported | - |
| Colchero et al., 2017 | Mexico |  | **/** | Quantitative | N/A | Household (purchasing data) | Household purchasing data of SSB, including information on food and beverages purchased from stores and socio-demographic variables | Nielsen Consumer Panel Services | City | S | Changes in purchases of taxed and untaxed beverages in the post-tax years were estimated using two separate models (regression model): comparing 2014 with predicted volumes (counterfactual) based on pre-tax (2012-2013) trends and comparing 2015 with the same counterfactual. | An average decline of -7.6% for the post-tax period was found. Untaxed beverages purchased increased 2.1% in the post-tax period. | - Incomplete data on dairy beverage purchases before October 2012 - Average purchases in household surveys tend to be underesti-mated | - |
| Zuleta et al., 2023 | Peru | **/** |  | Qualitative (Document review and interview) | N/A | Involved stakeholders: government (MOH, MOF), media, researchers, advocates, representative from company and trade association | 1. Media material  2. Government document  3. Stakeholder interview transcripts | 1. Database for news articles (LexisNexis and Factiva) 2. Transparency and Access to Public Information Law- No.28706 from Ministry of Economic and Finance 3. Semi-structured interviews | National | P,S | Thematic analysis was applied to identify arguments supporting and arguments against tax policy | - MOF is a leading actor in health tax (incl. SSB) implementation. They are independent from the Congress, there was not much political debate and lobbying in the Congress, and civil society is strong in supporting the action  - Arguments on health and economic support the movement of policy  - The main opposing actors include the alcohol, SSB, and tobacco industries, by using economic, trade-related arguments and criticizing the policy process | Limited access to government interviews and more information from industry | - |
| Dasco et al., 2023 | Philippines |  | **/** | Quantitative | N/A | All population groups | SSB consumption at household and individual levels in 2013, 2015 and 2018-19 | National Nutrition Survey | National | S | Consumption among households was collected from food weighing, food recall, and total intake of food items. Individual consumption data were collected from a 24-hour food recall. Data were weighted and analyzed using STATA to analyze descriptive statistics such as percentages and mean amount of SSB intake per day. | Intake of SSB at the household level was significantly lower in the 2018-19 survey, especially in carbonated drinks, sweetened powdered drinks and sweetened teas.  Intake of SSB at the individual level also declined from 2013 to 2018-19, ranging from 43%-57%, with the intake of sweetened juice drinks significantly declining the most in all age and population groups. | Recall bias | Study that assesses the impact of the tax on the purchasing power of sweetened beverages and other types of beverages, as well as a study on evaluating the impact of SSB on health |
| Smit et al., 2023 | South Africa |  | **/** | Quantitative | N/A | Dietitians and key industry role-players from government, the public sector and academia | Perspectives on the implementation of HPL (SSB tax included) | Cross-sectional survey | National | P | Data was collected via the online survey. The outcome measurement included awareness and opinions of SSB tax policy, perceived SSB purchasing of consumers and barriers or facilitators for implementation. Descriptive statistics were used for data analysis. | Although dietitians (97.7%; n = 127/130) were aware of the HPL only 29 (22.3%) dietitians knew that SSBs over 4 g sugar per 100 ml are taxed. Dietitians were positive towards the policy, although the majority agreed that the implementation of a sugar tax alone would not make a difference because multiple factors contribute to NCDs and obesity. The KIRs regarded the policy as insufficient to influence consumers’ purchasing behavior. KIRs felt that consumers’ habitual purchasing behavior creates a barrier to successful implementation. The main enabling factor was the educational level of the consumer. | - Samples cannot be generalized  - Bias from dietitians regarding their clients' self-report purchasing and consumption, not the general consumers' practice | Study to include questions to test knowledge on the SSB tax |
| Bercholz et al., 2022 | South Africa |  | **/** | Quantitative | N/A | Household (purchasing data) | Household purchase data from Jan 2014 to Mar 2019 among 3000 households, the data were weighted to cover 90-95% of the population. Purchasing data were merged from several sources to determine the nutritional content of purchased products | Europanel | National (weighted) | S | Data were analyzed by assessing the decomposing changes in sugar concentration and then the sugar content of SS, using the mathematical equation. Further analysis was on decomposing changes from sugar content to volume of beverage purchases. Analysis was done based on the beverage items rather than the individual products. | 1. Most of the beverage items were not reformulated, but for taxed products, lower sugar concentration increased from 5.2% before the implementation to 17.1% in the post-implementation period. 2. Sugar content from beverage purchases fell from 15.2 g/capita/day before the tax to 13.5 g/capita/day after its announcement, and to 10.4 g/capita/day after it came into effect, totaling a 4.9 g/capita/day overall decrease. | - It does not identify the causes of the observed reductions in the sugar content of beverage purchases; not only tax policy but also media campaigns play a role  - Could not identify substitute products | A study that explores how the consumer and producer factors compare in different segments of sugary drink consumption distribution (e.g. different income group) |
| Essman et al., 2022 | South Africa |  | **/** | Quantitative | N/A | Adults aged 18-39 living in a low-income town | SSB intake, knowledge on SSB, risk perception and tax awareness | Cross-sectional survey | City | P | Data were collected two months before tax implementation and a post-tax survey 12 months. 24-hour diet recalls were conducted by interviewers with nutrition training. Data were analyzed using a logistic regression model. | At post-tax, the predicted probability to consume taxed beverages was 33.5% for those who expressed an intention to reduce SSB intake compared to 45.9% for those who did not. SSB knowledge increased slightly, from 70.2% correct pre-tax to 72.3% correct post-tax. The tax effect on SSB intake was modified by SSB knowledge and intention to reduce SSB intake, with higher levels of each associated with lower SSB intake. | - Unable to make causal claims about the relationships between individual-level psychological constructs and participant behavior, and how the SSB tax affected these - Did not identify substitution effects | Study to find the causal claims about the relationship between individual-level psychological constructs and behavior |
| Koen et al., 2022 | South Africa |  | **/** | Quantitative | N/A | People aged more than 18, literate, doing 50% of the food shopping for the household | Awareness and understanding of the tax policy, opinions about the sugar tax, and purchasing behavior and sugar consumption | Cross-sectional survey (Interviewer-administered questionnaires) | City | P | The questionnaire consisted of socio-demographic information, lifestyle and behavior attributes, awareness and understanding of the tax policy, opinions about the sugar tax, and purchasing behavior and sugar consumption. Data was analyzed using STATISTICA with summary statistics and chi-square tests. Open-ended questions were categorized and key themes were identified. | Although most people (46%) were aware of tax policy, only 0.3% identified the correct amount of sugar allowed in SSBs before being levied. 54.5% noticed the price increase on SSB, and 43.1% of these changed their purchasing and consumption because SSB was expensive. | - Sample was not representative - Fatigue from participants and interviewers may affect the quality of data | Study includes the grocery stores for lower SES to increase the diversity of study participants |
| Ross et al., 2022 | South Africa |  | **/** | Quantitative | N/A | 1. Retail shop in the area  2. Adults aged 18-39 living in a low-income town | 1. Price of SSB, both taxed and untaxed, in retail outlets 2. SSB Purchasing in lower-income adults | Cross-sectional survey | City | P | 1. The price of SSB was collected before and after the implementation of the tax and compared by volume and store type. Data was analyzed using paired  t-test.  2. Survey included anthropometric measures, a household questionnaire assessing food acquisition, knowledge, attitudes, perceptions and behaviors and a 24-hour diet recall and beverage questionnaire. Logistic regression was used to estimate differences in weekly beverage acquisition before and after tax. | 1. Prices of taxed SSB increased significantly among small shops and supermarkets between 2018 and 2019. There were non-significant decreases in the price of untaxed beverages in small shops, but prices of untaxed beverages increased in supermarkets.  2. There was a 10-percentage-point decrease in purchasing regular soda in supermarkets, a 12-percentage-point decrease in small-owned shops, and no significant change in other independent stores. | - Bottled water was not captured  - Lack of geographic control or comparison city | Study to assess the cost shifting to see how retailers respond to the tax |
| Essman et al., 2021 | South Africa |  | **/** | Mixed method (Quantitative content analysis) | N/A | News media | News media content related to SSB tax | Nexis Uni and ProQuest Central | National | S | Online news articles were selected based on keywords related to SSB tax, published before the tax (2016) to after the tax (2019) period. Interested outcomes included health and economics, source attributed to the topic, support or opposition, and proposed solutions. Data were first analyzed for descriptive information to see the number of supportive and opposing, or balanced news articles. The data were coded for the health and economics statement. | 81% mentioned health, and 65% mentioned economics topics. 54% of articles expressed support and 26% expressed opposition to SSB tax policy. All sources except industry expressed majority support for the HPL. Health reasons were the most common justifications for support, and economic harms were the most common justifications for opposition. Industry (15%) or government (73%) were the actors most commonly proposed to fix the problem of overconsumption of sugar. | - Unable to make causal claims about the effects of the news media on how people respond to SSB taxes - Only English sources were included | Study that explores the linkage between news media exposure and SSB intake (to understand the impacts of news media on diet behaviors) |
| Kruger et al., 2021 | South Africa | **/** |  | Qualitative | Multiple Streams Theory | Academic, civil society, health organization, industry, industry association, industry-funded research organization, labor and other | Public statements, policy proposals and responses, submissions or bills, parliamentary committee minutes, public statements, events, and media coverage | Various sources including Parliament Monitoring Group Database, National Treasury website, and Melwater Media Monitoring | National | S | The study applied the Multiple Streams Theory for policy adoption and implementation analysis. Data were collected from 2002-2020 from various sources. Documents were analyzed based on stakeholders and their sentiment (supportive, opposed, or neutral) to the tax policy. | - After passing the Bill, the media was more focused on the negative effects on the sugar industry on job losses  - Industry and labor reported negative impacts of the tax in the media before and after the implementation period - Media reported an increase in revenue after tax implementation, and some was used for a public health campaign - SSB producers introduced a range of reformulated or diet products - Civil society organizations supported the increase of tax to 20% in 2020, but it did not occur - Negative news articles on tax after implementation were from the sugar industry, where the concerns on imported cheap sugar, duty-free, and decreasing sugar prices were reported and could be a result of the failure of the tax revision in the 2020-21 fiscal year. | - Data were based on document review, no primary data collection  - Only publicly available documents were included | - |
| Stacey et al., 2019 | South Africa |  | **/** | Quantitative | N/A | N/A | Retail prices | Statistics South Africa's Consumer Price Index (CPI) | National | S | 1. Pre-post analysis (regression analysis) used for price change  2. Using data on price change of carbonated products to estimate pass-through rate (regression analysis) 3. For carbonated products where the sugar content was reduced, not reduced, and reduced below 4g/100 ml, further analyzed of reformulation and price change (regression analysis) | 1. An average 1.006 ZAR increase in price per liter on all carbonated beverages after tax implementation  2. The overall pass-through rate was 68% 3. Price increase was found in the brands that were reformulated (reduced sugar content) | No counterfactual effect analysis | Study in a large firm to analyze intra-firm heterogeneity in price response |
| Essman et al., 2018 | South Africa |  | **/** | Quantitative | N/A | Adults aged 18-39 living in a low-income town | SSB consumption | Cross-sectional survey | City | P | Data collected 2 months before and 12 months after tax, using a 24-hour recall. Outcome variables included intake of total sugar, energy, and volume of SSB from taxed, untaxed and substituted products were analyzed using a 2-part model. Nutrition composition was analyzed using nutrition facts panel (NFP) data. | Among taxed beverages, sugar intake decreased significantly from 28.8 g/capita/day pre-tax to 19.8 g/capita/day post-tax. Energy intake decreased from 121 kcal/capita/day pre-tax to 82 post-tax. Volume intake decreased from 315 ml/capita/day pre-tax to 198 post-tax. The opposite trend was found in untaxed beverages. | - Social desirability bias could have affected reporting and caused us to underesti-mate SSB intake - Data could not be generalized to the high-income group | Study to explore the responses to the SSB tax from different SES status |
| Phulkerd et al., 2022 | Thailand |  | **/** | Quantitative | N/A | Thai people aged 15 years and above | SSB consumption, sociodemographic, lifestyle behavior | Longitudinal Survey | National | P | Data were from the second wave (2019) of the ‘Longitudinal Monitoring Survey of Sugar-Sweetened Beverage Consumption’, the first wave was conducted in 2018. Consumption/non-consumption of SSB and average daily amount of SSB consumed were the main variables to measure taxed SSB consumption. Logistic regression was employed to find the association of consumption and sociodemographic characteristics and health-related factors. | Carbonated drinks were consumed the most among participants (225 ml/person/day). Sociodemographic characteristics (male, residing in Bangkok or Central region, lower educational attainment), physical inactivity, and smoking were associated with a higher level of SSB consumption. There was also association between lack of knowledge of recommended limit on sugar intake and a higher level of carbonated drinks consumption. | - Recall bias  - Only taxed SSB consumption was observed, not non-taxed or locally made included | Study to include other types of SSB apart from taxed SSB, such as non-taxed and locally-made SSB, in particular issues such as perception of SSB tax, and consumer price effects of taxed SSB |
| Phulkerd et al., 2020 | Thailand |  | **/** | Quantitative | N/A | Thai people aged 15 years and above | SSB consumption | Longitudinal Survey | National | P | Data were collected in 2018 and 2019. Participants reported weekly frequency and daily servings of taxed and non-taxed SSBs. Descriptive statistics assessed consumption changes, and t-tests compared differences between taxed and non-taxed SSB consumption. between taxed SSB and non-taxed SSB consumption. | Average daily SSB consumption declined from 474.0 mL in 2018 to 453.8 mL in 2019, or a 2.5% decrease. All age groups reported reduced SSB consumption. There was a reduction in both taxed and non-taxed SSBs consumed between 2018 and 2019, with −2.8% and −2.0%, respectively. | - Self reported data, recall bias  - Lack of substitute behavior | - Study to assess the manufacturers responses to the SSB tax (both taxed and non-taxed products) - Study to assess the maintenance of behavioral changes and their purchasing, product substitution, reformulation, and health status |
